# Effects of physical form of *β*-lactoglobulin and calcium ingestion on GLP-1 secretion, gastric emptying and energy intake in humans: a randomised crossover trial

**DOI:** 10.1017/S0007114524000321

**Published:** 2024-05-28

**Authors:** Jonathan D. Watkins, Harry A. Smith, Aaron Hengist, Søren B. Nielsen, Ulla Ramer Mikkelsen, John Saunders, Francoise Koumanov, James A. Betts, Javier T. Gonzalez

**Affiliations:** 1 Centre for Nutrition, Exercise and Metabolism, Department for Health, University of Bath, Bath, UK; 2 Arla Foods Ingredients Group P/S, Viby J, Denmark; 3 Royal United Hospital, Bath, UK

**Keywords:** Calcium, Protein, GLP-1, Appetite, Energy intake

## Abstract

The aim of this study was to assess whether adding Ca^2+^ to aggregate or native forms of *β*-lactoglobulin alters gut hormone secretion, gastric emptying rates and energy intake in healthy men and women. Fifteen healthy adults (mean ± s d: 9M/6F, age: 24 ± 5 years) completed four trials in a randomised, double-blind, crossover design. Participants consumed test drinks consisting of 30 g of *β*-lactoglobulin in a native form with (NATIVE + MINERALS) and without (NATIVE) a Ca^2+^-rich mineral supplement and in an aggregated form both with (AGGREG + MINERALS) and without the mineral supplement (AGGREG). Arterialised blood was sampled for 120 min postprandially to determine gut hormone concentrations. Gastric emptying was determined using ^13^C-acetate and ^13^C-octanoate, and energy intake was assessed with an *ad libitum* meal at 120 min. A protein × mineral interaction effect was observed for total glucagon-like peptide-1 (GLP-1_TOTAL_) incremental AUC (iAUC; *P* < 0·01), whereby MINERALS + AGGREG increased GLP-1_TOTAL_ iAUC to a greater extent than AGGREG (1882 ± 603 *v*. 1550 ± 456 pmol·l^−1^·120 min, *P* < 0·01), but MINERALS + NATIVE did not meaningfully alter the GLP-1 iAUC compared with NATIVE (1669 ± 547 *v*. 1844 ± 550 pmol·l^−1^·120 min, *P* = 0·09). A protein × minerals interaction effect was also observed for gastric emptying half-life (*P* < 0·01) whereby MINERALS + NATIVE increased gastric emptying half-life compared with NATIVE (83 ± 14 *v*. 71 ± 8 min, *P* < 0·01), whereas no meaningful differences were observed between MINERALS + AGGREG *v*. AGGREG (*P* = 0·70). These did not result in any meaningful changes in energy intake (protein × minerals interaction, *P* = 0·06). These data suggest that the potential for Ca^2+^ to stimulate GLP-1 secretion at moderate protein doses may depend on protein form. This study was registered at clinicaltrials.gov (NCT04659902).

Glucagon-like peptide-1 (GLP-1) and peptide tyrosine-tyrosine (PYY) are gut hormones produced by intestinal L-cells^(1,2)^. Whilst GLP-1 stimulates glucose-dependent insulin secretion^(3,4)^, both GLP-1 and PYY delay gastric emptying^(5,6)^ and reduce food intake^(7,8)^. These actions can contribute to improved metabolic health, such that GLP-1 agonism has been targeted for obesity and type 2 diabetes management^(9)^. Strategies including the administration of GLP-1 agonists and dipeptidyl peptidase-IV inhibitors (DPP4; which rapidly cleaves GLP-1 upon binding) have been effective at promoting weight loss and greater glycaemic control in patients with obesity or type 2 diabetes, respectively^(10,11)^. Moreover, one mechanism proposed for the dramatic weight loss and diabetic remission associated with bariatric surgery is increased gut hormone availability^(12,13)^. However, many of these approaches are either expensive and/or carry risks of unwanted side effects, so additional strategies to increase gut hormone availability are still sought as alternatives or adjunct to pharmacological approaches.

Nutrition potently regulates enteroendocrine cell action and subsequent peptide hormone release through nutrient sensing and absorption^(14,15)^. Different receptors related to GLP-1 secretion may be targeted by specific macro/micronutrient intake^(16)^. One such receptor, the Ca^2+^-sensing receptor (CaSR), has been implicated in gut hormone release evidenced by *in vitro* ^(17,18)^, *ex vivo* ^(19)^ and *in vivo* experiments in rodent models^(20)^. The CaSR is a class C G-protein-coupled receptor that is responsive to extracellular Ca (principal ligand) and amino acids/peptides^(21,22)^ and is expressed in intestinal L-cells^(23)^. Human studies, however, have provided conflicting results regarding the effects of protein-Ca^2+^ co-ingestion on gut hormone secretion. GLP-1 secretion was enhanced by about 25 % when Ca is added to a large (50 g) bolus of whey protein hydrolysate in lean individuals^(24)^, but Ca did not increase GLP-1 secretion when added to a more moderate dose of a whey protein hydrolysate (25 g) in individuals with overweight/obesity^(25)^. The nature of these conflicting data could be due to several factors, including population, protein dose and/or protein form.

*β*-Lactoglobulin is the major protein in whey protein isolate. Under denaturing conditions, *β*-lactoglobulin can unfold from its native form and subsequently form aggregates and gel networks. When aggregates of *β*-lactoglobulin produced by thermal aggregation are subjected to acidification in the stomach, they are prone to further association near the isoelectric point of about 5·2 to form gel structures^(26,27)^. This at least in part occurs by inducing the formation of intermolecular bridges between chains, *via* shielding of negatively charged carboxylic groups^(28)^. Furthermore, Ca^2+^ is thought to modulate the heterogeneity of the microstructure formed by *β*-lactoglobulin aggregates which may affect digestive properties^(29,30)^. Since gelation of glucose solutions via the use of gel fibres can slow gastric emptying rates and decrease energy intake^(31,32)^, it could be speculated that gelation of *β*-lactoglobulin in the acidic environment of the stomach may also slow gastric emptying rates, with effects on gut hormone availability, appetite and energy intake.

Accordingly, the primary aim of this study was to assess gut hormone secretion and gastric emptying rates in humans following ingestion of *β*-lactoglobulin in native and aggregate form, both with and without co-ingestion of Ca^2+^. It was hypothesised that Ca^2+^ co-ingestion would potentiate gut hormone secretion and slow gastric emptying rates, but that these effects would be more pronounced with the aggregate form of *β*-lactoglobulin than the native form.

## Methods

### Experimental design

To establish whether the form of *β*-lactoglobulin influences the ability of Ca to stimulate GLP-1 secretion and gastric emptying, we employed human experiments with postprandial blood and breath sampling. A Ca-rich milk mineral supplement was used as the vehicle for providing Ca and phosphate on the basis that Ca phosphate may display specific effects on the structural integrity of protein aggregates, providing greater external validity.

Fifteen metabolically healthy men and women (Table 1) were recruited to participate in a double-blinded randomised crossover study with four trial arms. Inclusion criteria included age: 18–65 y, BMI: 18·5–25·0 kg·m^−2^, no history of metabolic or gastrointestinal disease, and free from allergies or intolerances to Ca or milk proteins. Following written consent, participants were randomly assigned to a trial sequence by a researcher who was not involved in data collection. The study protocol was approved by the NHS Central Bristol Research Ethics Committee (20/SW/060; IRAS: 277805). All procedures were carried out in accordance with the latest version of the Declaration of Helsinki. The study was registered at clinicaltrials.gov (NCT04659902).

**Table 1.** Participant characteristics

	Total		Males		Females	
	Means	sd	Means	sd	Means	sd
Sample size (*n*)	15		9		6	
Age (years)	24	5	26	5	21	4
Body mass (kg)	71·3	10·1	77·1	5·5	62·6	9·4
BMI (kg·m^−2^)	22·7	1·8	23·3	1·3	21·8	2·1

Data are means ± sd.

### Pre-trial standardisation

Participants recorded their diet using a paper food diary for 24 h prior to trial 1 and then replicated this diet for 24 h prior to each subsequent trial. Participants were asked to eat habitually although refrain from caffeine, alcohol and any vigorous unaccustomed physical activity for 24 h prior to a trial day. Participants were provided with a standardised meal (Tesco spinach and ricotta cannelloni 440 g; 463 kcal, 56 g carbohydrate, 10 g sugars, 25 g fat and 24 g protein), which was consumed no later than 22.00 the evening before a trial day. A gluten-free alternative standardised meal (Amy’s Kitchen vegetable lasagne 255 g; 360 kcal, 41 g carbohydrate, 6 g sugars, 15 g fat and 15 g protein) was provided for one participant. In an effort to reflect real-life conditions, women were tested at different phases of their menstrual cycle, that is, menstrual cycle was not standardised within or between participants. While we acknowledge this could be considered a limitation, to check if this influenced the overall inferences of the study, sensitivity analysis was performed with females included *v*. removed from the analyses (there was no indication of differential responses). The washout period between trials was between 2 and 7 d.

### Trial days

Participants arrived at the laboratory between 09.00 and 10.00 h following a 10–14 h overnight fast (standardised within participants) having only consumed about 473 ml (pint) of water between waking and attending the laboratory. Water consumption was permitted *ad libitum* and recorded on first condition and replicated on the subsequent trials. Height was measured using a stadiometer (Seca Ltd), with participants barefoot in the Frankfurt plane. Body mass was measured using digital scales (Tanita) with participants barefoot and wearing light clothing.

Arterialised blood samples were obtained by retrograde cannulation of a pre-heated dorsal hand vein. One participant was cannulated in the antecubital vein which was still arterialised by heating the arm, and sensitivity analysis indicated that this did not influence the overall inference. Site was then matched within participants. A subset of participants (*n* 3) was fitted with a nasogastric tube by a qualified researcher. The nasogastric tube was roughly 0·5 cm wide and passed about 60 cm down in the stomach from the nostrils. Litmus paper was used to check the correct position of the tube. To assess whether the use of a nasogastric tube influenced the responses, sensitivity analysis was performed with *v*. without these three participants in the overall dataset, and the inferences remained unaltered.

**Fig. 1.**
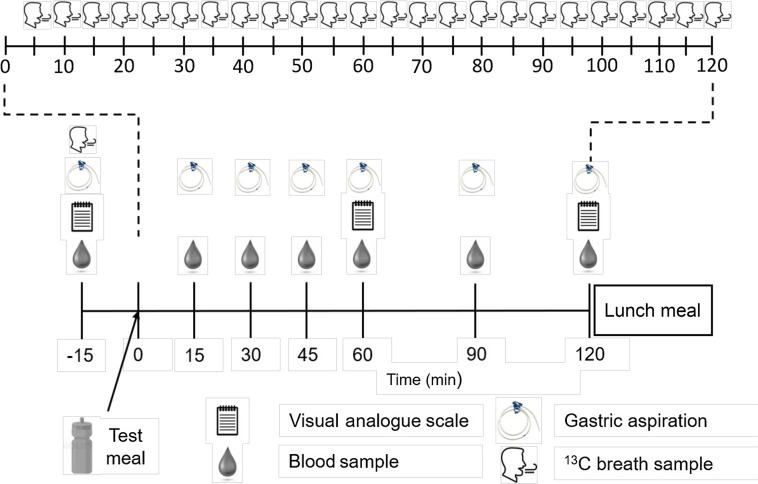
Schematic of trial days.

Baseline measures of blood, breath, visual analogue scales (VAS) of appetite and, if applicable, gastric aspirate were collected prior to the test meal. Test solutions were consumed within a 5-min window (trial commenced upon the first mouthful of the test solution). The time taken to ingest the test solution on trial 1 was recorded and replicated for each subsequent trial. Following the ingestion of the test solution, a palatability and blinding validation scale were completed. Breath samples for the quantification of gastric emptying were collected every 5 min, and blood samples were taken at 15, 30, 45, 60, 90, and 120-min following consumption of the test solutions. VAS was obtained at 60 and 120 min. If applicable, gastric aspirate from the stomach was sampled at every time point at which blood was sampled. At the end of this period, participants ingested an *ad libitum* lunch (Tesco Hearty Food Co. cheese and tomato pasta 400 g; nutritional info per 100 g, 118 kcal, 20 g carbohydrate, 16 g sugars, 7 g fat and 14 g protein) in isolation, free from distractions, until they were comfortably full. Meals were prepared in a large bowl and replenished every about 5 min so that the contents of the bowl never completely emptied. A gluten-free alternative (Amy’s Kitchen mac and cheese 255 g; nutritional info per 100 g, 181 kcal, 23 g carbohydrate, 2 g sugars, 7 g fat and 6 g protein) was provided for one participant (the inclusion/removal of this participant did not influence the overall statistical inferences). This was followed by another VAS and palatability scale. Upon the completion of the final trial, participants completed a restrained eating questionnaire. The protocol is outlined in Fig. 1.

### Test solutions

There were four test solutions consumed in a randomised order (researchrandomizer.org; JG) over four trials: (1) *β*-lactoglobulin (derived from whey protein isolate) in a native form (NATIVE) (32 g providing 30 g protein; Lacprodan BLG 100 Neutral (Arla Foods Ingredients, Viby J)); (2) NATIVE plus milk minerals rich in Ca (NATIVE + MINERALS) (9547 mg to provide 2497 mg Ca; Capolac® MM-0525 BG (containing calcium phosphate, Arla Foods Ingredients, Viby J)); (3) *β*-lactoglobulin in an aggregate form (AGGREG) (500 ml to provide about 30 g protein (Arla Foods Ingredients, Viby J)); and (4) AGGREG plus milk minerals rich in Ca (AGGREG + MINERALS) (9547 mg to provide 2497 mg Ca; Capolac®). The Ca content in the local tap water was 99·58 mg·l^−1(33)^, and day-to-day variation is < 15 mg which is unlikely to alter any responses measured^(24)^. Ca content was matched to that of the highest doses in our previous study^(25)^. Thirty litres of 6 % AGGREG pH 7·0 was prepared by hydration of NATIVE powder in 25 litres of demineralised water followed by pH adjustment to 7·0 using 7 % NaOH and final adjustment with demineralised water to 30 litres. Aggregates were produced during ultra-high temperature treatment at 143°C for 4 s using a plate heat exchanger after which the sample was cooled to 10°C and tapped into sterile bottles.

Test solutions were prepared by two researchers not involved with data collection. Each drink contained 80 mg of low-energy-sweetened chocolate flavouring (My Protein) and was labelled with 150 mg (1–^13^C)-sodium acetate and 100 mg (1–^13^C)-sodium octanoate for the measurement of gastric emptying. Five hundred millilitres of water were added to NATIVE and NATIVE + MINERALS to volume match the test solutions. The composition of test solutions is given in Table 2.

**Table 2.** Nutritional composition of each 500 ml test solution for each condition

Ingredient	Condition			
	NATIVE*	NATIVE + MINERALS†	AGGREG‡	AGGREG + MINERALS§
Energy (kJ)	498	515	498	515
Energy (kcal)	119	123	119	123
Water (ml)	500	500	500	500
Sucralose (mg)	80	80	80	80
Ca (mg)||	65	2612	56	2603
P (mg)	15	1065	25	1075
Mg (mg)	<0·1	67	25	92
Protein (g)	30	30	30	30
Carbohydrate (g)	<0·1	0·8	<0·1	0·8
Fat (g)	<0·1	<0·1	<0·1	<0·1

* NATIVE, *β*-lactoglobulin derived from whey protein isolate, native form

†NATIVE + MINERALS, NATIVE with Capolac^®^.

‡AGGREG, *β*-lactoglobulin derived from whey protein isolate, aggregate form

§AGGREG + MINERALS, AGGREG with Capolac^®^.

||Reported Ca value includes the estimated tap water content based on 50 mg of Ca per 500 ml^(33)^.

### Blood sampling and analysis

A 5-ml sample of arterialised venous blood was collected at each time point. This was dispensed into 5 ml of EDTA collection tubes (Sarstedt) treated with 2500 KIU aprotinin (Sigma-Aldrich Company Ltd) to inhibit DPP4 activity. The tube underwent immediate centrifugation at 4000 g for 10 min at 4°C, and the plasma was transferred to new tubes and stored on dry ice for the remainder of the trial. Plasma samples were stored at −80°C until analysis.

Plasma GLP-1_TOTAL_ (EZGLP1T-36), GLP-1_ACTIVE_ (EZGLPHS-35K), PYY_TOTAL_ (EZHPYYT66K), DPP4 (DC260B) and insulin (EZHI-14K) were measured using commercially available ELISA (Merck Millipore, except DPP4 which was from R&D systems). The antibodies in the GLP-1_TOTAL_ assay detect both GLP-1_7–36amide_ and GLP-1_9–36amide_, whereas the antibodies in the GLP-1_ACTIVE_ are specific to GLP-1_7–36amide_ only. The GLP-1_TOTAL_ assay is therefore indicative of total secretion, whereas the GLP-1_ACTIVE_ assay is more indicative of hormone action^(34)^. Similarly, the antibodies in the PYY_TOTAL_ are specific to both PYY_1–36_ and PYY_3–36_. DPP4 ELISA plates failed to detect any DPP4 in the samples of five participants, which limited the sample size for this outcome (*n* 10). Total amino acid concentrations for plasma samples were measured using an L-amino acid quantification assay (Merck Millipore). All samples for comparison between treatments within each participant were included on the same plate, and the respective intra-plate CV for GLP-1_TOTAL_, GLP-1_7–36amide_, PYY_TOTAL_, Insulin and total amino acids were 10·1 %, 6·8 %, 9·9 %, 9·6 % and 7·3 %, respectively. It is acknowledged that a CV > 10 % is a limitation of this study.

### Subjective appetite ratings

Subjective ratings of appetite were assessed using a previously validated 100-mm VAS^(35)^. The four questions from this scale: ‘How hungry do you feel?’, ‘How full do you feel?’, ‘How satisfied do you feel?’ and ‘How much do you think you can eat?’ were converted into a composite appetite score. This score combines hunger, fullness, satisfaction and prospective consumption using the following equation^(36)^:

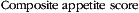




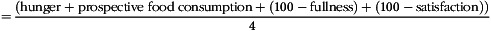




### Gastric emptying and aspiration

Five ml stomach aspirate was sampled at baseline, 30, 60 and 120 min, and aliquoted appropriately for analyte profiling. The (1–^13^C)-acetate/octanoate technique was employed to measure both the liquid and semi-solid phases of gastric emptying as previously described^(37,38)^. Each test solution was labelled with 150 mg (1–^13^C)-sodium acetate and 100 mg (1–^13^C)-sodium octanoate. Breath samples were then collected every 5 min after the ingestion of the liquid test meal and were analysed for isotopic enrichment by an isotope ratio mass spectrometer (Iso-Analytical) with an online gas chromatographic purification system. All δ values were expressed *v*. the Pee Dee Belemnite international standard and related to the baseline value. This was converted to the percentage (^13^C) recovery per hour and to the cumulative recovery after 2 h, which acts as a marker of gastric emptying^(38,39)^.

### Statistical analysis

The primary outcome was plasma GLP-1_TOTAL_ incremental AUC (iAUC). Gastric emptying, *in vitro* GLP-1_TOTAL_ secretion, concentrations of plasma GLP-1_7–36amide_, PYY_TOTAL_, DPP4, insulin and plasma amino acid profiles were secondary outcomes, alongside VAS and *ad libitum* energy intake. The sample size calculation was based on our previous data^(24)^: the mean difference for postprandial plasma GLP-1_TOTAL_ between the co-ingestion of whey protein and Capolac® *v*. whey protein alone was 9·1 ± 6·9 pmol·l^−1^ × 120 min (mean ± sd). Using this effect size (*d* = 1·32), fifteen participants would provide about 85 % probability (power: 0·86) of detecting such an effect with an α-level of 0·05 using a one-way, repeated-measures ANOVA with two tails. Data are presented as means ± sd in text and means ± 95 % CI in figures. The iAUC or total AUC (tAUC) was calculated for all variables (other than *ad libitum* energy intake and *in vitro* GLP-1_TOTAL_) using the Time Series Response Analyser^(40)^.

Time series data were compared by three-way (time × protein form × minerals) repeated-measures ANOVA. Summary data (e.g. iAUC) were compared by two-way (protein form × minerals) repeated-measures ANOVA. ANOVA statistical tests are considered robust to violations to normality assumptions^(41)^. Where an interaction effect was determined, *post hoc* Bonferroni adjustments were applied for multiple comparisons. A cubic model was used to determine gastric emptying half-times on the basis that this provided an *r* ^2^ of > 0·996. A two-tailed *P* value of ≤ 0·05 was deemed statistically significant. Results were analysed using Microsoft Excel version 16.0, SPSS statistical software version 25.0 and GraphPad Prism version 8.4.3.

## Results

### Plasma glucagon-like peptide-1 and peptide tyrosine-tyrosine concentrations

**Fig. 2.**
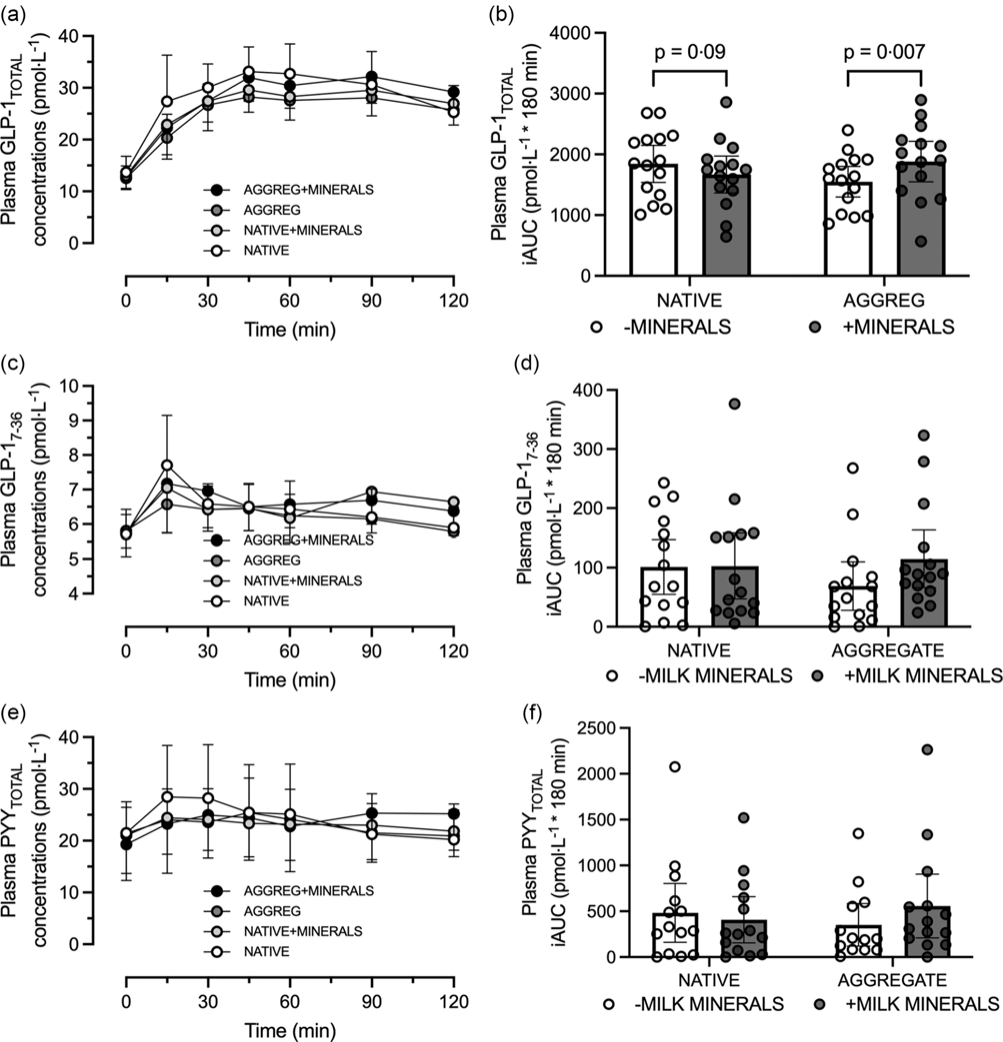
Plasma GLP-1_TOTAL_ concentrations (a), GLP-1_TOTAL_ iAUC (b), GLP-1_7–36_ concentrations (c), GLP-1_7–36_ iAUC (d), PYY_TOTAL_ concentrations (e) and PYY_TOTAL_ iAUC (f) following consumption of 30 g *β*-lactoglobulin in native form (NATIVE) or aggregate form (AGGREG) with (+MINERALS) and without (-MINERALS) Ca-rich milk minerals. Data are means ± 95 % CI. *n* 15. GLP-1, glucagon-like peptide-1; iAUC, incremental AUC; PYY, peptide tyrosine-tyrosine.

For plasma GLP-1_TOTAL_ concentrations, no minerals × protein form × time interaction was detected (*P* = 0·06; Fig. 2(a)), although the GLP-1_TOTAL_ iAUC did display a minerals × protein form interaction effect (*P* = 0·002; Fig. 2(b)), whereby the GLP-1 _TOTAL_ iAUC was higher MINERALS + AGGREG compared with AGGREG (*P* < 0·01), but not with MINERALS + NATIVE compared with NATIVE (*P* = 0·09).

A minerals × protein form × time interaction was detected for GLP-1_7–36_ concentrations (*P* = 0·05; Fig. 2(c)), although *post hoc* comparisons were not significant following adjustment for multiple comparisons. Similarly, no minerals × protein form interaction effect was detected for the GLP-1_7–36_ iAUC (*P* = 0·18; Fig. 2(d)).

Similarly, no minerals × protein form × time interaction was detected for PYY_TOTAL_ concentrations (*P* = 0·51; Fig. 2(e)) and no minerals × protein interaction for PYY_TOTAL_ iAUC (*P* = 0·31; Fig. 2(f)).

### Plasma insulin, amino acid and dipeptidyl peptidase-IV concentrations

**Fig. 3.**
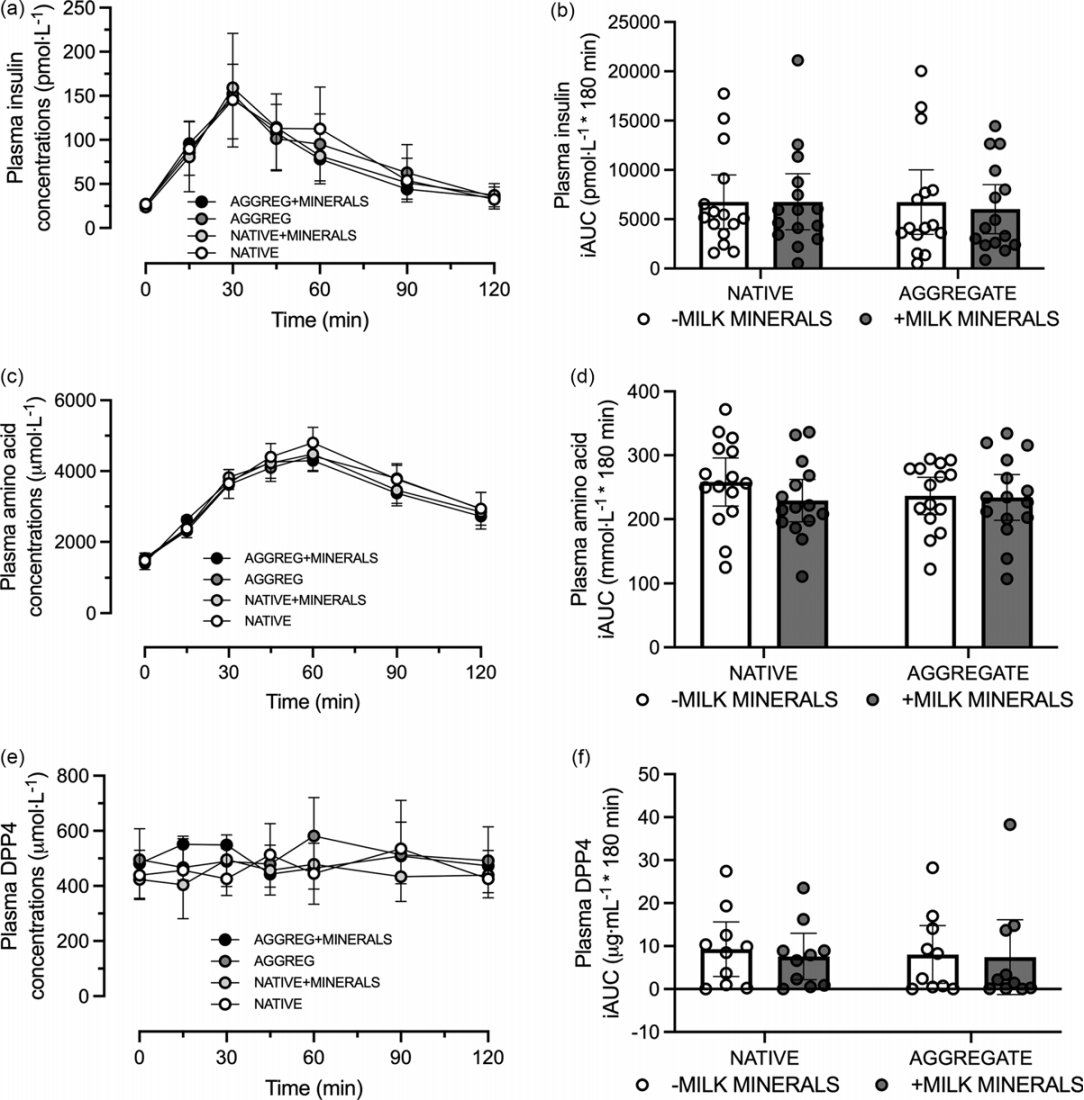
Plasma insulin concentrations (a), insulin iAUC (b), amino acid concentrations (c), amino acid iAUC (d), PYY_TOTAL_ concentrations (e) and PYY_TOTAL_ iAUC (f) following consumption of 30 g *β*-lactoglobulin in native form (NATIVE) or aggregate form (AGGREG) with (+MINERALS) and without (-MINERALS) Ca-rich milk minerals. Data are means ± 95 % CI. *n* 15. iAUC, incremental AUC; PYY, peptide tyrosine-tyrosine.

No minerals × protein form × time interactions were detected for concentrations of insulin, amino acids or DPP4 (*P* = 0·8, 0·3 and 0·4, respectively; Fig. 3). Similarly, the iAUC for insulin, amino acids and DPP4 did not display a minerals × protein form interaction (*P* = 0·45, 0·17 and 0·9, respectively; Fig. 3).

### Gastric emptying and gastric pH

**Fig. 4.**
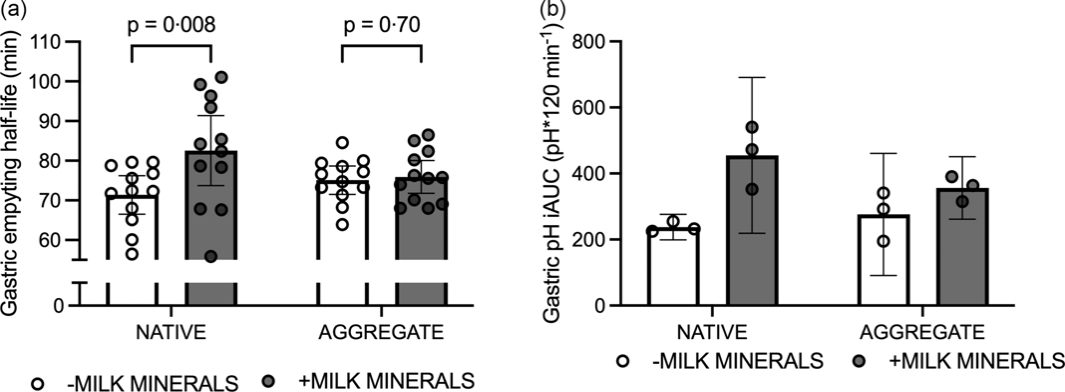
Gastric emptying half-life iAUC determined by ^13^C-acetate and ^13^C-octanoate breath tests (a) and gastric pH iAUC (b) following consumption of 30 g *β*-lactoglobulin in native form (NATIVE) or aggregate form (AGGREG) with (+MINERALS) and without (-MINERALS) Ca-rich milk minerals. Data are means ± 95 % CI. *n* 15 for panel a and 3 for panel b. iAUC, incremental AUC.

Gastric emptying half-life displayed a min effect of minerals (*P* < 0·01), whereby addition of minerals increased gastric emptying half-life. In addition, a minerals × protein form interaction was detected (*P* = 0·03), whereby MINERALS + NATIVE increased gastric emptying half-life compared with NATIVE (*P* < 0·01; Fig. 4(a)), whereas no such effect was observed between MINERALS + AGGREG compared with AGGREG (*P* = 0·7). Gastric pH demonstrated a similar response in the subgroup of *n* 3, whereby a main effect of minerals was detected (*P* = 0·02), where addition of minerals increased pH iAUC, although a minerals × protein interaction was not observed (*P* = 0·17; Fig. 4(b)).

### Appetite ratings and energy intake

**Fig. 5.**
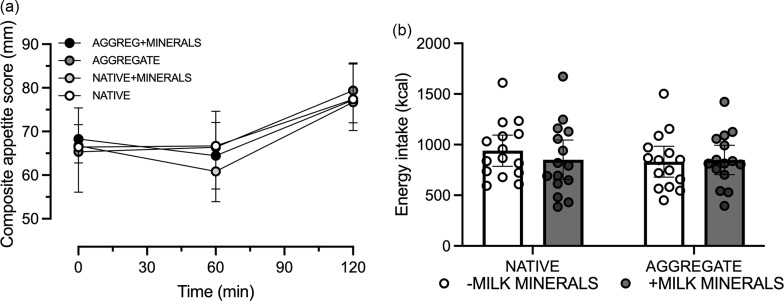
Compositive appetite score (a) and *ad libitum* energy intake (b) following consumption of 30 g *β*-lactoglobulin in native form (NATIVE) or aggregate form (AGGREG) with (+MINERALS) and without (-MINERALS) Ca-rich milk minerals. Data are means ± 95 % CI. *n* 15 for panel a and 3 for panel b. iAUC, incremental AUC.

The composite appetite score did not display a minerals × protein form × time interaction effect (*P* = 0·6; Fig. 5(a)). However, energy intake did display a main effect of protein form (*P* = 0·02), whereby AGGREG tended to lower energy intake compared with NATIVE, albeit a minerals × protein form interaction effect was not detected (*P* = 0·06; Fig. 5(b)).

### Standardisation and blinding

The test solutions were well tolerated by all participants and were correctly identified on 19 % of occasions, with 33 % of participants unable to identify a single drink correctly. There was no difference in the time taken to consume the test solutions between conditions (*P =* 0·18; NATIVE (283 ± 137 s); NATIVE + MINERALS (273 ± 118 s); AGGREG (268 ± 120 s); AGGREG + MINERALS (279 ± 131 s). Data were checked for order effects, and the only parameter to exhibit any evidence of systematic variance between repeated trials was *ad libitum* energy intake (*P* = 0·002). *Ad libitum* energy intake (kcal) was lower in trial 1 compared with all other trials (all *P* < 0·05). Although, there was no trial order × condition interaction detected (*P* = 0·06).

## Discussion

In the present study, postprandial GLP-1 secretion was modestly increased by the co-ingestion of Ca-rich milk minerals (about 2600 mg Ca) with the aggregate form of *β*-lactoglobulin (about 30 g), but no such effect was seen when Ca was added to the native form of *β*-lactoglobulin. There were no differences between conditions in plasma GLP-1_7–36amide_ or PYY_TOTAL_, nor were there any differences in plasma insulin, total amino acids or DPP4 between conditions. Both stable isotope and pH measures suggested that addition of Ca could slow gastric emptying rates, which was seen most clearly when added to the native form of *β*-lactoglobulin. These changes in GLP-1 and gastric emptying did not clearly translate into a change in appetite ratings or energy intake.

Interestingly, in the present study, GLP-1_TOTAL_ iAUC was only enhanced by Ca when added to the aggregate form of *β*-lactoglobulin and not the native form. Previous work has demonstrated enhanced GLP-1 concentrations following meals supplemented with Ca in comparison with the same meals without Ca^(42–44)^, and the combination of Ca and protein/amino acids has been shown to stimulate GLP-1, *in vivo* ^(24)^ and *ex vivo* ^(19)^ to a greater extent than protein/amino acids alone. Despite this, in our previous work, co-ingestion of whey protein hydrolysate and Ca enhanced GLP-1_TOTAL_ iAUC in comparison with whey protein hydrolysate alone on one occasion,^(24)^ but not the other^(25)^. These contrasting findings may be due to the dose and/or type of protein ingested, in addition to other macronutrients. The observed effect following perfusion of L-phenylalanine and Ca through rodent small intestine on GLP-1 secretion was observed at supraphysiological concentrations of 10 mM for 90 min^(19)^. In humans, the data from the present study and others suggest that any effect of Ca on further stimulating GLP-1 secretion with whey protein may require protein doses above 30 g. This may be due to the rate of protein digestion, whereby ingesting above 30 g protein in a bolus leads to greater amino acid accumulation in the intestine^(45)^ to allow interactions with Ca on L-cells. Interestingly, Ca did further stimulate GLP-1 secretion with only 30 g of protein, when the *β*-lactoglobulin was in aggregate form. In the present study, the difference observed in GLP-1_TOTAL_ iAUC was not sustained for GLP-1_ACTIVE_ iAUC, and this may be explained by a couple of reasons. First, it is well known that GLP-1_ACTIVE_ is degraded at a rapid rate, and because of this, it is possible that the detection threshold was not consistently reached by the time GLP-1_ACTIVE_ was sampled in the arm. This is supported by the greater variance in GLP-1_ACTIVE_ compared with GLP-1_TOTAL_. Second, there is a possibility that a type 2 error has been committed. Overall, these findings suggest that not only protein dose, but also protein form may modulate the effects of Ca-protein co-ingestion on GLP-1 secretion.

It was expected that the aggregate form of *β*-lactoglobulin would be susceptible to gelation in the acidic environment of the stomach and that this may be modulated by Ca. In turn, this could delay gastric emptying rates. In contrast to this hypothesis, we saw no effect of Ca co-ingestion on gastric emptying rates when added to AGGREG. Ca did, however, delay gastric emptying rates when added to NATIVE. Bile salts and salts of fatty acids with high affinity for Ca have previously been shown to delay gastric emptying rates due to shrinking of lateral intracellular spaces^(46)^, and it may be that as the aggregate form of protein already had a marginally slow gastric emptying rate as a baseline, the addition of Ca did not further slow this process. Another possibility is that gelation did not alter gastric emptying rates. Although, we cannot confirm whether gelation did occur as this was not measured, and the absence of such gelation could explain the lack of effect on gastric emptying. It may be expected that slowing gastric emptying rates would decrease amino acid absorption and availability. However, we did not observe any meaningful differences between conditions in the postprandial rise amino acid concentrations. It is possible that gastric emptying is not rate-limiting to amino acid availability with the doses of protein used in the current study. These data also suggest that effects of Ca co-ingested with protein on gastric emptying and energy intake are independent of GLP-1, PYY or amino acid availability.

In addition to gastric emptying and intestinal amino acid availability as hypothesised ways to manipulate endogenous GLP-1 availability, whey proteins have also been demonstrated as sources of DPP4 inhibitors^(47)^. DPP4 inhibition can reduce the degradation of GLP-1_7–36_, thereby maintaining a high concentration of receptor-active GLP-1^(10)^. In the present study, we saw no effect of protein form or Ca co-ingestion on DPP4 concentrations, nor any clear, meaningful effects on GLP-1_7–36_ concentrations. It is therefore unlikely that the form of *β*-lactoglobulin alters DPP4 availability or activity in the circulation.

Unlike GLP-1, the addition of Ca to AGGREG did not enhance PYY_TOTAL_, which is surprising considering that both hormones are similarly released from intestinal L-cells^(2)^. Increasing Ca dose augmented PYY release similarly to GLP-1 *ex vivo* ^(19)^, but Ca co-ingestion with protein has not been shown to stimulate PYY release to a greater extent than protein alone in humans^(24)^. In male Wistar rats, intraduodenal L-tryptophan administration with the CaSR antagonist NPS 2143 suppressed GLP-1 release in comparison with L-tryptophan alone, but this was not the case for PYY, suggesting that the CaSR is not involved in mediating PYY secretion^(48)^. Despite this, in isolated loops of rat small intestine, perfusion of amino acids in the presence of NPS 2143 significantly reduced total PYY AUC in comparison to without NPS 2143^(19)^. Furthermore, in mucosal L-cells, PYY-Y1 activation (a PYY receptor) partially mediates an increase in glutamine induced electrical L-cell activity, which is dampened following co-administration of NPS 2143^(49)^. These findings suggest the involvement of CaSR in protein-mediated PYY release is inconclusive and, as of yet, protein and Ca co-ingestion has failed to translate to enhanced PYY release in humans.

Importantly, while an increase in GLP-1_TOTAL_ iAUC with the addition of Ca to the aggregate form was observed, it must also be considered whether this difference is physiologically meaningful and/or has practical relevance. In a previous meta-analysis, a mean GLP-1 infusion rate of just 0·89 pmol·kg^−1^·min^−1^ across seven studies (infusions over 0–240 min, mean 60 min) led to a reduction in *ad libitum* energy intake of about 206 kcal (13·2 %) and about 60 kcal (4·5 %) in lean and overweight individuals, respectively, in comparison with a saline infusion^(50)^. By contrast, the mean difference in GLP-1_TOTAL_ iAUC following ingestion of the aggregate form with or without Ca was about 2·8 ± 4·7 pmol·l^−1^·min^−1^. Therefore, the observed difference may not have been sufficient to affect energy intake in the current study, and higher protein doses may be needed for Ca to exert such an effect^(24)^.

In the present study, *ad libitum* energy intake was not influenced by the addition of Ca to either form of protein, suggesting the difference in GLP-1_TOTAL_ iAUC between these conditions did not translate into any change in energy intake. It should be noted that the sample size was based on the GLP-1_TOTAL_ iAUC, and therefore it is possible that the sample size was too low to detect small effects in energy intake and other variables (e.g. GLP-1_7–36_ and PYY_TOTAL_). Furthermore, the difference in energy intake observed between protein forms should be considered with some caution. An effect of trial order was observed whereby energy intake was systematically lower in the first trial completed compared with each other trial. Interestingly, the aggregate form without Ca was randomly assigned as the first trial on 6/15 occasions (i.e. with four conditions and fifteen participants, it is impossible to fully counterbalance for every possible permutation of sequences). To determine whether this influenced energy intake, an additional statistical test was conducted to determine whether there was an interaction between trial order and condition. There was no condition × trial order interaction effect. Overall, the data in this study did not provide evidence that addition of Ca to either native or aggregate forms of *β*-lactoglobulin alters energy intake despite detectable effects on GLP-1 and gastric emptying rates. In addition to effects on energy intake, GLP-1 can increase glucose-stimulated insulin secretion. We did not observe differences in insulin concentrations between conditions, although this may not be surprising given that the test drinks did not contain any appreciable quantities of carbohydrate.

In conclusion, postprandial concentrations of GLP-1_TOTAL_ were potentiated by the addition of Ca-rich milk minerals to an aggregate form of *β*-lactoglobulin but not when added to the native form. In contrast, gastric emptying rates were decreased by the addition of Ca-rich milk minerals to a native form of *β*-lactoglobulin. These data suggest that, in addition to protein dose, the form of protein can also modulate the effectiveness of Ca on further stimulating postprandial GLP-1 secretion.
